# Neutralizing monoclonal antibody against Dickkopf2 impairs lung cancer progression *via* activating NK cells

**DOI:** 10.1038/s41420-019-0204-4

**Published:** 2019-07-31

**Authors:** Tianli Shen, Zhengxi Chen, Ju Qiao, Xuejun Sun, Qian Xiao

**Affiliations:** 1 Department of General Surgery, First Affiliated Hospital of Xi’an Jiaotong University, Xi’an, Shaanxi China; 2 Department of Pharmacology, School of Medicine, Yale University, 10 Amistad St, New Haven, CT USA; 3 Department of Orthodontics, Shanghai Ninth People׳s Hospital, School of Stomatology, Shanghai key Laboratory of Stomatology, Shanghai Jiao Tong University, Shanghai, China; 4 Department of Mechanical and Industrial Engineering, Northeastern University, Boston, MA USA

**Keywords:** Tumour immunology, Lung cancer

## Abstract

Adenomatous polyposis coli (*APC*) and *KRAS* proto-oncogene (*KRAS*) mutations frequently co-occur in non-small cell lung cancer. Inactivating *APC* mutations in colorectal carcinoma has been well characterized, leading to the approaches targeting on dysregulated *APC* pathway. However, it remains undetermined whether such approaches are also applicable to non-small cell lung cancer patients harboring similar mutations of *APC*. Dickkopf-related protein 2 (DKK2) is a Wnt antagonist. Our previous study has proved that anti-DKK2 antibody 5F8 suppressed the growth of colorectal carcinoma with *APC* mutations, illustrating a new target agent of APC-mutated tumors. This study aimed to investigate the potential of applying anti-DKK2 antibody to non-small cell lung cancer with *APC* mutations. We found significant upregulation of Dkk2 expression in *APC*-mutated lung cancers. Administration of DKK2 antibody inhibited cancer growth *via* modulating tumor immune microenvironment in lung cancer mouse models. Our study provided strong evidence supporting *APC* mutations-directed applications of anti-DKK2 targeted therapy in a wide range of cancer types, including lung cancer.

## Introduction

Lung cancer is one of the leading causes of cancer-related death, resulting in more than 1 million deaths worldwide annually^1,2^. Lung cancer can be histologically classified into four major categories: lung adenocarcinoma, squamous cell carcinoma (SCC), large cell carcinoma, comprising non-small cell lung cancer (NSCLC), and small cell^3–9^ carcinoma of the lung. Among these categories, NSCLC is more common (~80%). The top recurrent mutated genes in NSCLC include *KRAS* proto-oncogene (*KRAS*). It was reported that activating *KRAS* mutations were present in about 4% of SCC and 30% of adenocarcinoma^10,11^.

In human, a wide range of cellular processes are under the control of Wnt signaling pathways^12–17^. Briefly, the canonical Wnt/β-catenin pathway is initiated by two types of cell-surface receptors: low-density lipoprotein receptor-related proteins 5 and 6 (LRP5 and LRP6, respectively), and Frizzled proteins^18^. Dysregulation of Wnt signaling contributes to many human diseases, including cancer^12–15^. Adenomatous polyposis coli (*APC*) is one of the key components in canonical Wnt/β-catenin pathway^19,20^. *APC* mutations, primarily nonsense mutations and frameshift insertions and deletions encoding truncated proteins, were subsequently identified in a majority of sporadic colorectal adenomas and carcinomas^21,22^. Somatic *APC* mutations have furthermore been described in several other solid tumors, most prominently in gastric and pancreatic cancers^22–24^. In 2004, *APC* mutations have been reported in lung NSCLC and small-cell lung carcinoma^25^. Interestingly, *APC* mutations in lung cancer co-occurred with *KRAS* mutations in NSCLC, including adenocarcinoma and SCCs^26^.

Direct pharmacological strategies targeting *KRAS*-driven cancers are not clinically available^27,28^. However, the fact that *APC* and *KRAS* mutations frequently co-occur in NSCLC provides therapeutic opportunities to target altered *APC* pathway in such group of patients. Oncogenic *APC* mutations have been well characterized in colorectal carcinoma because they are dominant mutations that drive the development of colorectal cancer. Numerous therapeutically strategies targeting altered Wnt pathway in *APC* mutant colorectal cancers are under preclinical and clinical studies, including ours^29^. These approaches are potentially applicable to lung cancer patients harboring inactivating mutations of *APC*^28^.

Dickkopf-related protein 2 (DKK2)^14,30^, one of the Wnt antagonists, inhibits Wnt-β-catenin signaling by binding to LRP5 and LRP6^31^. DKK2 has a less critical role in vertebrate development and adult life^32–34^. DKK2 deficiency leads to decreased blood glucose^35^ and a moderate reduction in bone mass^32^. In our recent study, we uncovered a function of DKK2 in promoting tumor progression by suppressing cytotoxic immune cell activation in colorectal carcinoma with *APC* mutations^29^. The loss of *APC* in intestinal tumor cells upregulated the expression of DKK2 through β-catenin pathway, which, together with its receptor LRP5, provided an unconventional mechanism for tumor immune evasion. The monoclonal antibody-mediated ablation of DKK2 impeded tumor progression and enhanced the effects of PD-1 blockade. This antibody presented a promising therapeutic effect on *APC*-mutated colorectal cancer^29^.

In the present study, we applied anti-DKK2 antibody to lung cancer mouse models driven by *APC* mutation. We found that anti-DKK2 antibody suppressed the tumor growth. We also found significant upregulation of Dkk2 expression in human lung cancer tissues with *APC* mutations. Taken together, we illustrated a new targeted strategy for NSCLC with *APC* mutations.

## Results

### Upregulation of DKK2 expression in human NSCLC with *Apc* mutation

**Fig. 1:**
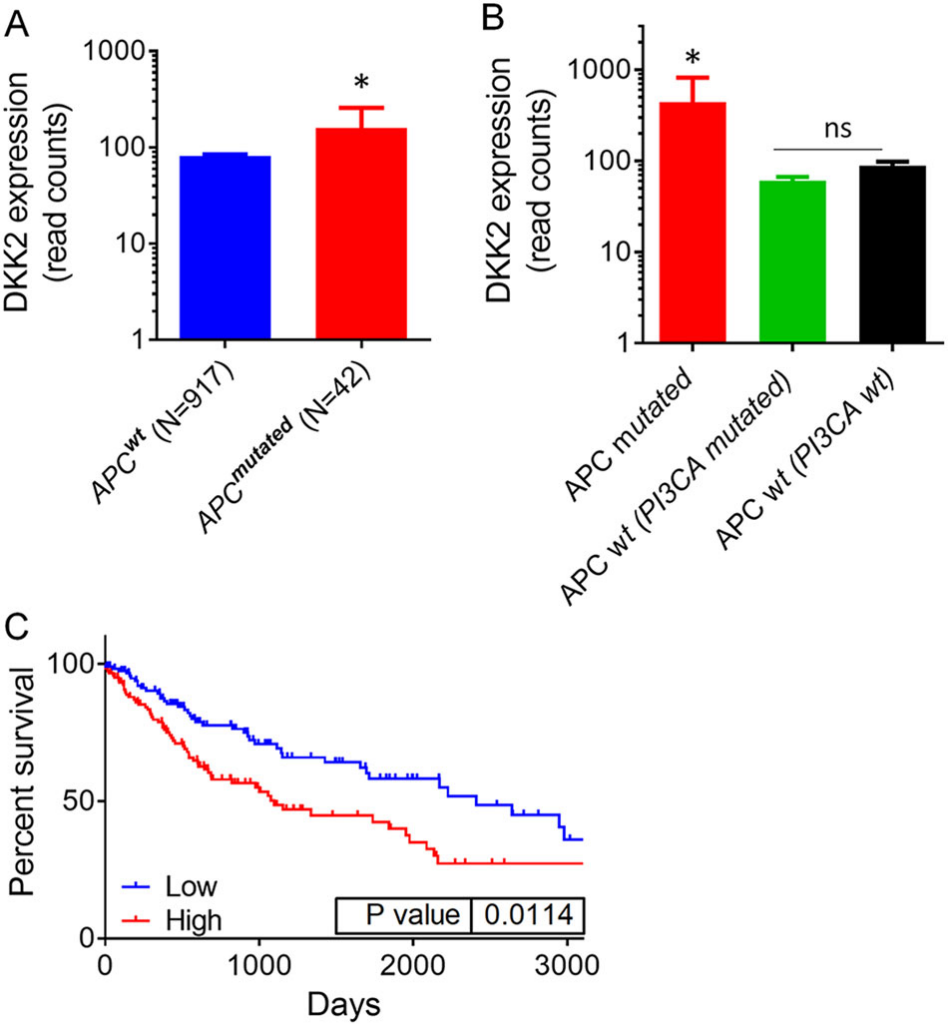
High DKK2 expression is present in human non-small cell lung cancers with *APC* mutation and predicts poor survival. **a** Analysis of DKK2 expression in *APC*^*wt*^, and *APC*^*mutated*^ non-small cell lung cancer groups. Data on DKK2 expression were obtained from the TCGA data sets from OncoLnc, then separate them into two groups according to the mutation status of APC. APC mutation includes 9 Deep Deletion, 17 Truncating Mutation, 34 Missense Mutation, and 1 Inframe Mutation. DKK2 read counts are RSEM RNA SeqV2 normalized. **b** Further divided *APC*^*wt*^ group into two groups according to mutation status of PI3CA mutation in lung squamous cell carcinoma. **c** Analysis of the correlation of DKK2 expression and survival. Data on DKK2 expression, overall survival were obtained from the TCGA data sets from OncoLnc. The high and low DKK2 expressers were grouped on the basis an arbitrary cutoff percentile of 25%. Mantel–Cox log-rank tests were done using the GraphPad Prism 7 software. (*p < 0.05; **p < 0.01)

Data on *Dkk2* RNA expression of NSCLC were obtained from the TCGA data sets from OncoLnc. Two groups were separated according to the mutation status of *APC*: *APC*^*wt*^, and *APC*^*mutated*^ groups. *APC* mutation includes 9 deep deletion, 17 truncating mutation, 34 missense mutation, and 1 inframe mutation. Upregulation of *Dkk2* expression is found in *APC*^*mutated*^ group compared with *APC*^*wt*^ group (Fig. 1a). This suggests that DKK2 might have an important role in these NSCLCs with *APC* mutation. Our previous data indicated that gain-of-function mutations of *PI3K* also is linked to elevated *Dkk2* expression^29^. Then, the *APC*^*wt*^ group was further divided into *APC*^*wt*^ with mutated PI3CA, and *APC*^*wt*^ with wt PI3CA in lung SCC. The RNA level of *Dkk2* is only associated with *APC* mutation status, excluding the PI3CA mutation status (Fig. 1b). Furthermore, analysis of the TCGA data sets showed that high *Dkk2* expression correlates with poor survival rates (Fig. 1c). These results indicated loss of *APC* upregulate the expression of DKK2 in human NSCLC.

### DKK2 is highly upregulated in *Kras*^*LSL-G12D*^; *Apc*^*fl/fl*^ mouse lung tumor tissues

**Fig. 2:**
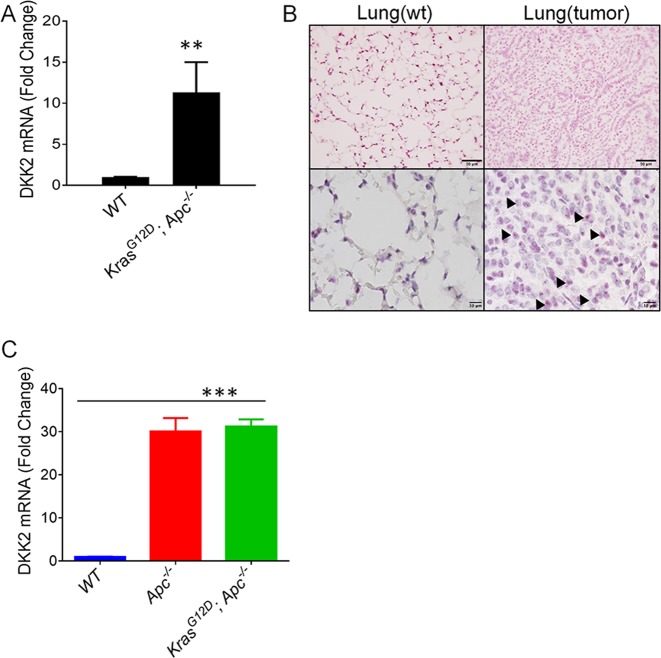
Upregulation of DKK2 expression in *Kras*^*G12D*^; *Apc*^*−/−*^ mouse lung tumor tissue. **a** DKK2 RNA isolated from lung tumors from *Kras*^*G12D*^; *Apc*^−*/−*^ and normal lung tissue from *WT* mice, respectively. DKK2 mRNA levels were determined by quantitative RT-PCR (data are presented as means ± sem; two-sided Student's *t* test, *n* = 3). **b** In situ hybridization to detect Dkk2 RNA expression is in lung cancer tissues and normal lung tissue. Arrow pointed DKK2 expression. Scale bars, 50 μm (top)/10 μm (bottom). **c** Dkk2 expression is upregulated by loss of APC in tumor organoids. Tumor organoids were isolated from *WT*, *Apc*^*−/*−^, and *Kras*^*G12D*^; *Apc*^−/−^ mice (data are presented as means ± sem; two-sided Student's *t* test, *n* = 2). (*p < 0.05; **p < 0.01; ***p < 0.001)

After *Kras*^*LSL-G12D*^; *Apc*^*fl/fl*^ mice were generated, we administrate Cre adenovirus by nasal injection to induce mouse lung cancer progression. Lung tissues from *WT* and *Kras*^*LSL-G12D*^; *Apc*^*fl/fl*^ were harvested for RNA extraction. Real-time PCR showed that *Dkk2* expression was significantly upregulated in mouse lung cancer compared with normal lung tissue (Fig. 2a). Concordantly, in situ hybridization confirmed that *Dkk2* expression is present in lung cancer tissues (Fig. 2b). We also isolated normal tissue or tumor organoids from *WT*, *Apc*^*fl/fl*^, and *Kras*^*LSL-G12D*^; *Apc*^*fl/fl*^ mice, and performed real-time PCR. These results also indicated that loss of *APC* upregulates *Dkk2* expression (Fig. 2c). Therefore, these results together supported the conclusion that *APC* loss drives DKK2 expression in both mouse and human NSCLC cells.

### Knockdown of DKK2 does not alter cell proliferation or apoptosis of LLC lung cancer cell in vitro

**Fig. 3:**
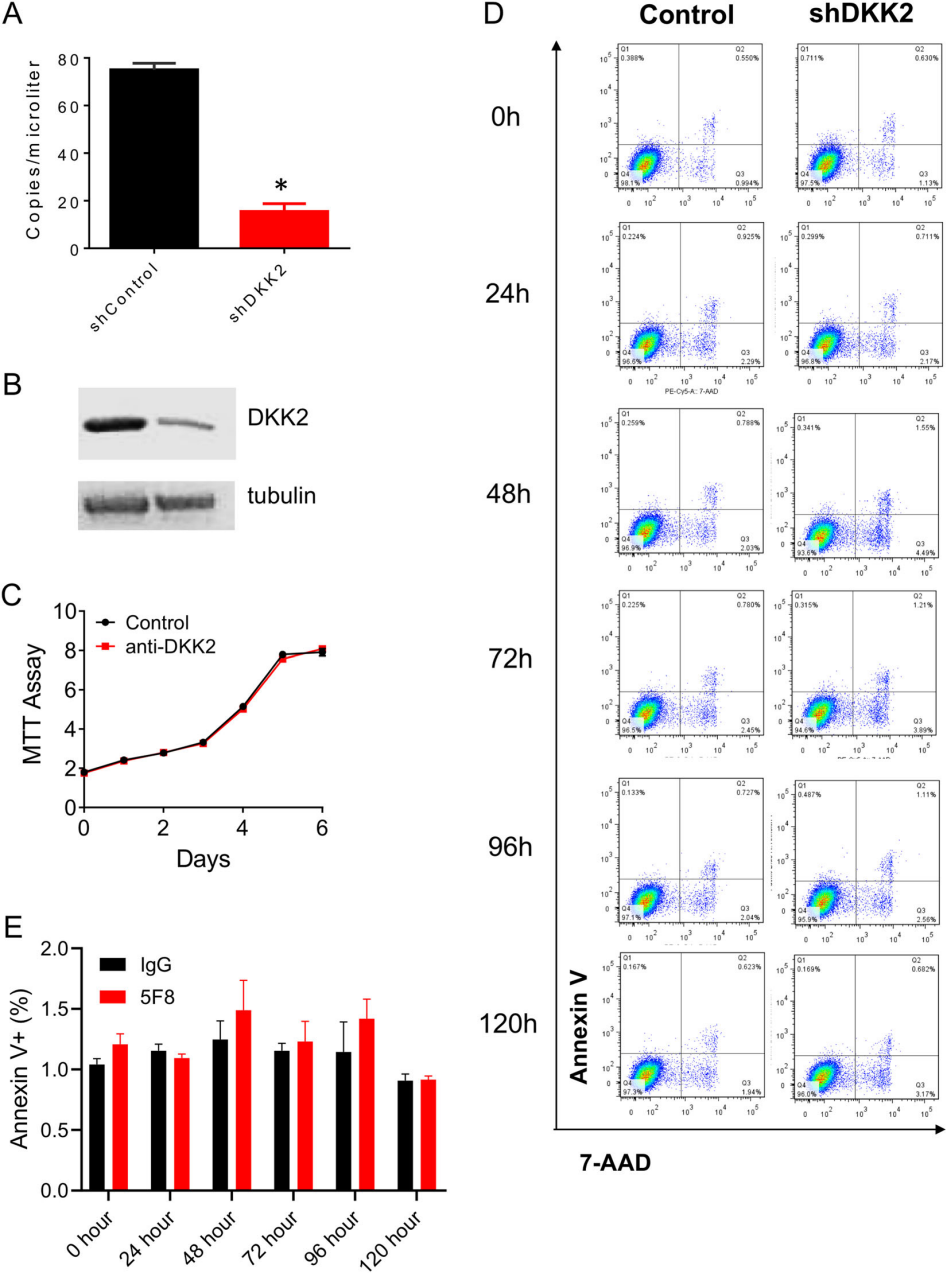
DKK2 knockdown does not affect cell growth and apoptosis. **a** DKK2 expression in LLC stable knockdown cells. DKK2 mRNA expression was determined by QuantStudio™ 3D Digital PCR System (data are presented as means ± sem; unpaired Student's *t* test, *n* = 3) using RNAs isolated from LLC cells with or without the shRNA. **b** Western analysis of DKK2 and tubulin levels were also shown. **c** MTT assays were performed to detect cell growth in LLC shscramble and shDKK2 cells. **d** Representative flow cytometry of 7-ADD and annexin V staining showed that there was no difference of cell apoptosis in shscramble and shDKK2 cells. **e** Quantification of annexin V+ cells. Data are presented as mean ± s.e.m. (*p < 0.05; **p < 0.01)

To investigate the role of DKK2 in lung cancer progression, we generated *Dkk2* knockdown stable cell lines. QuantStudio™ 3D Digital PCR showed more than 80% knockdown efficiency in LLC stable cell lines (Fig. 3a), and western blot further confirmed that DKK2 protein level was downregulated (Fig. 3b). Next, we determined cell growth and apoptosis. MTT assay showed that there was no difference in cell growth after *Dkk2* gene knockdown (Fig. 3c). Flow cytometry also indicated that knockdown of *Dkk2* has no effect on cell apoptosis (Fig. 3d, e). These findings are consistent with our previous studies in colon cancer cell lines^29^, DKK2 does not affect tumor cell itself proliferation and apoptosis.

### DKK2 knockdown inhibits tumor progression in vivo

**Fig. 4:**
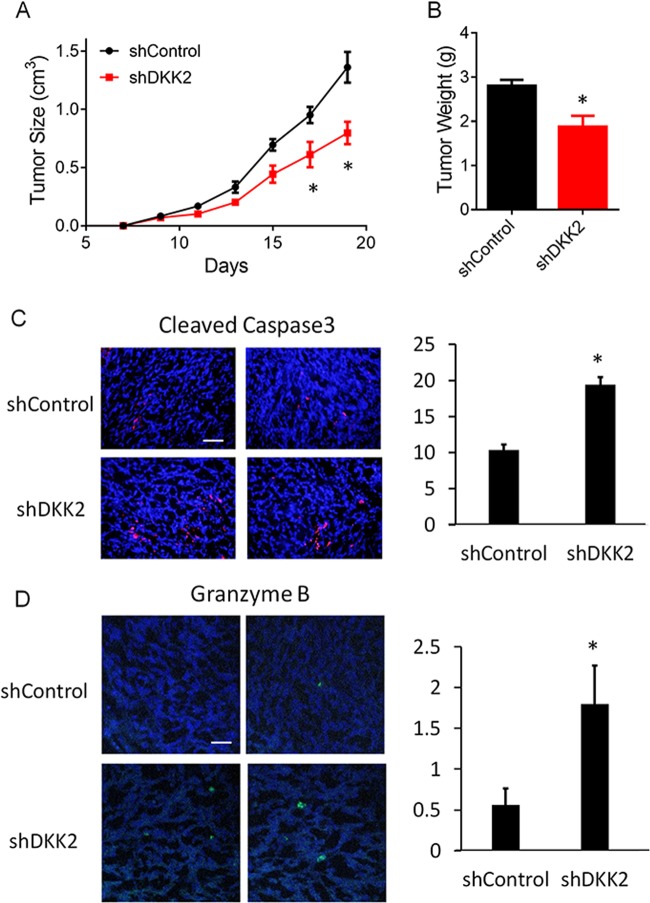
LLC orthotopic tumor model on *C57/BL* mice with or without DKK2 knockdown. **a**, **b** *C57/BL* mice inoculated with LLC shControl and shDKK2 cells. Tumors were collected at day 19 and weighed (*P* < 0.05 for tumor growth at days 17 and 19, respectively; two-way ANOVA; two-tailed Student’s *t* test for tumor weight; *n* = 5). **c** Effects of DKK2 knockdown on tumor apoptosis by staining of the sections of cleaved caspase3. The sections were also counterstained with DAPI. Five independent sections per tumor and five tumors per group were examined (unpaired Student’s *t* test). Scale bars, 100 μm. Quantification of cleaved caspase3 staining was shown (right). Data are presented as mean ± s.e.m. **d** Granzyme B staining in tumor sections from shControl and shDKK2 groups. The sections were also counterstained with DAPI. Five independent sections per tumor and five tumors per group were examined (unpaired Student’s *t* test). Scale bars, 100 μm. Quantification of granzyme B staining was shown (right). Data are presented as mean ± s.e.m. (*p < 0.05; **p < 0.01)

To study the effect of DKK2 on tumor progression in vivo, we performed tumor orthotopic experiment. Tumor cells with control shRNA or *Dkk2* shRNA were implanted into flank of *C57/BL* mice. We monitored tumor size every other day, and found that the tumor burden was decreased dramatically in *Dkk2* knockdown group (Fig. 4a). Tumor weight was also measured at the end-point of the day, which showed about 1/3 decrease in tumor mass (Fig. 4b). Because knockdown *Dkk2* did not affect the growth of LCC cells in in vitro culture, the impaired tumor progression in the shDKK2 group might alter tumor microenvironment. Immunofluorescence staining of the LLC tumors revealed that DKK2 knockdown significantly increased the number of apoptotic cells (Fig. 4c). The cleaved caspase3 protein is a member of the cysteine-aspartic acid protease family^36,37^. Sequential activation of caspase3 plays a central role in the execution phase of cell apoptosis^36,37^. The cytotoxic granzyme B (GrB)/perforin pathway has been traditionally viewed as a primary mechanism that is used by cytotoxic lymphocytes to eliminate tumor cells^36,37^. Accordingly, we observed that *Dkk2* knockdown increased the number of GZMB+ cells (Fig. 4d). These results implied that DKK2 may regulate the tumor immune microenvironment.

### DKK2 antibody administration inhibited lung cancer growth in vivo

**Fig. 5:**
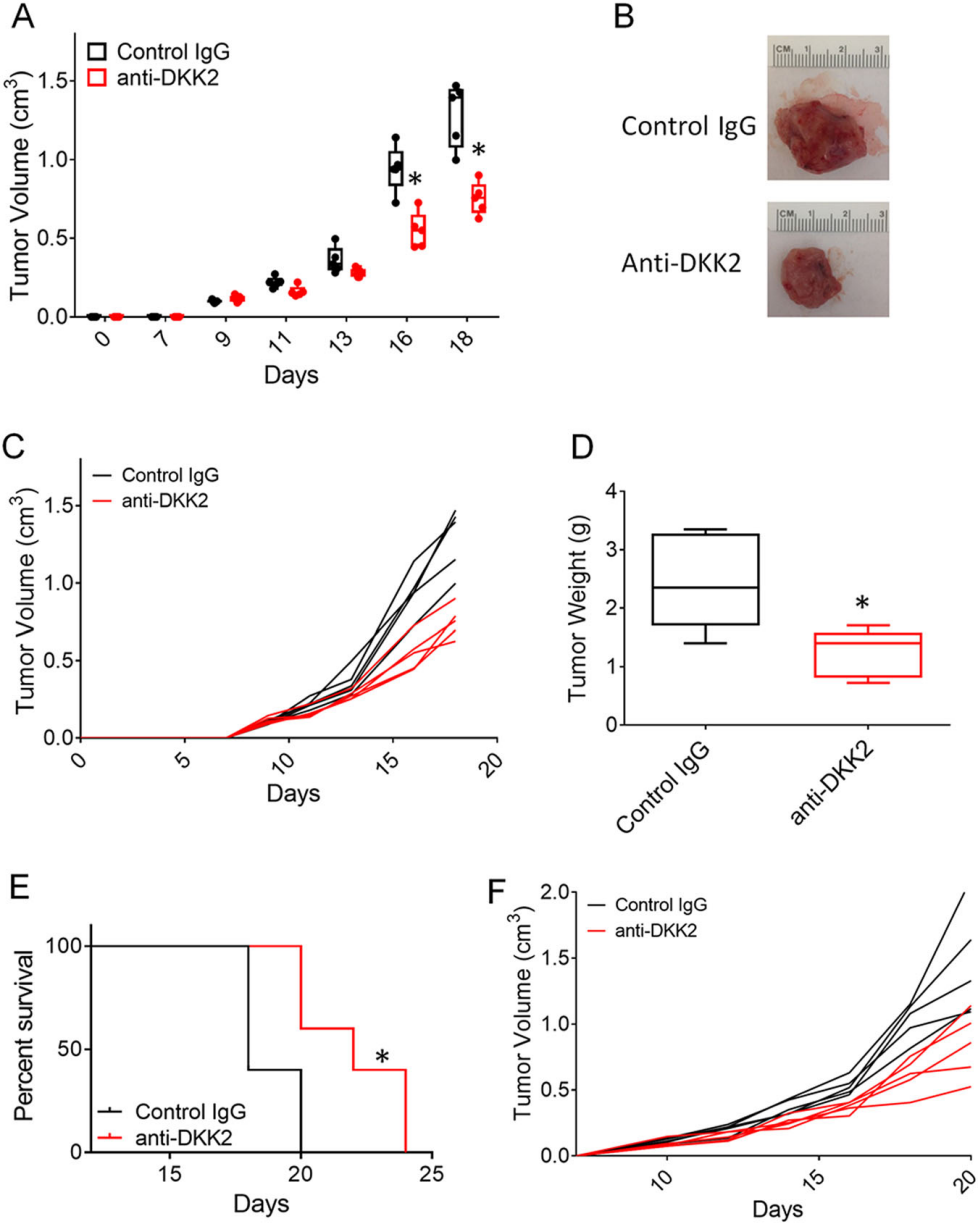
DKK2 antibody 5F8 treatment inhibited tumor growth on LLC orthotopic tumor model. **a**–**c** C57/BL mice inoculated with LLC cells were treated with anti-DKK2 antibody (10 mg/kg i.p. every other day) starting at day 13 after inoculation. Tumors were collected at day 18 and weighed (*P* < 0.05 for tumor growth at days 16 and 18, respectively; two-way ANOVA; two-tailed Student’s *t* test for tumor weight; *n* = 5). **a** Tumor size were presented as groups of IgG control and anti-DKK2 antibody. Individual data points represent individual tumors. **b** Representative picture of tumor at end-point time. Individual tumor growth was shown as **c**. **d** Tumor weight at day 18. **e** Survival evaluation of C57/BL mice inoculated with LLC cells were treated with anti-DKK2 antibody (10 mg/kg i.p. every other day) starting at day 10 after inoculation. (*P* < 0.01 for anti-DKK2 antibody treated versus IgG-treated; two-sided log-rank Mantel–Cox test; *n* = 5). Individual tumor growth was shown as **f**. (*p < 0.05; **p < 0.01)

We have generated a specific monoclonal antibody (5F8) targeting DKK2 protein, which has been proved that efficiently impaired tumor progression in both colorectal cancer and melanomas^29^. In this study, we would like to test its antitumor effect in lung cancer mice models. The mice bearing LLC tumor cells were treated with control IgG or anti-DKK2 antibody every other day when tumor approached 200 mm^3^. Constant with previous study^29^, DKK2 blockade significantly reduced tumor burden (Fig. 5a–c). Tumor weight was cut down by 50% compared with control IgG treatment group (Fig. 5d). We also observed that the administration of anti-DKK2 antibody extended the survival of tumor-bearing mice in the LLC tumor model (Fig. 5e, f). These data supported that DKK2 could promote tumor progression.

### DKK2 blockade modulates tumor immune microenvironment

**Fig. 6:**
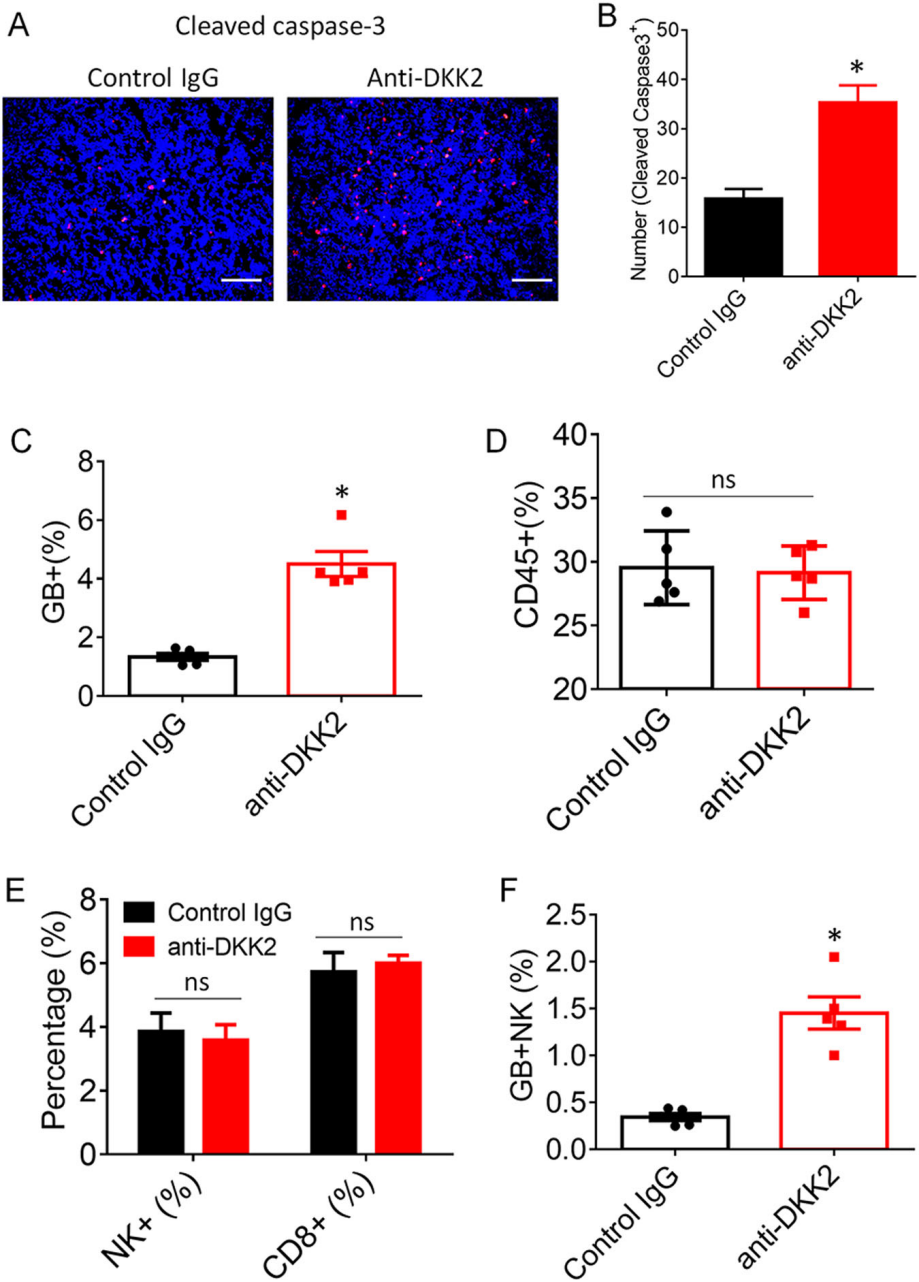
DKK2 antibody 5F8 treatment modulates tumor immune microenvironment. **a** Effects of anti-DKK2 treatment on tumor apoptosis, and number of cleaved caspase3+ cells were evaluated by staining of the sections of tumor for cleaved caspase3. The sections were also counterstained with DAPI. Five independent sections per tumor and five tumors per group were examined (unpaired Student’s *t* test). Data are presented as mean ± s.e.m. All experiments were repeated twice. Scale bars, 100 μm. **c**–**f** LLC cells were injected subcutaneously into C57BL/6 mice. When tumors reached 600 mm^3^ on average, the mice were given one injection of antibody (10 mg/kg i.p.). Tumors were collected 24 h later for flow cytometry analysis (two-tailed Student’s *t* test; *n* = 5). Tumor-infiltrating lymphocytes are pregated as CD45+ cell from total tumor population. **c** Quantification of the percentage of granzyme B+ cells in tumors from IgG or 5F8-treated tumors. **d** Quantification of the percentage of CD45+ TILs in tumors from IgG or 5F8-treated tumors. **e** Quantification of the percentage of infiltrated CD8+ and NK1.1+ cells in LLC tumors from IgG or 5F8-treated tumors based on flow cytometry analysis. **f** Quantification of the percentage of infiltrated GB+; NK1.1+ cells. Data are presented as means ± sem (ns, not significant; *n* = 5; two-sided Student *t* test). (*p < 0.05; **p < 0.01)

Furthermore, we performed the immunofluorescence staining of cleaved caspase3, which showed that the apoptotic cell numbers were increased to about three folds in anti-DKK2 antibody group, compared with those tumors treated with control IgG (Fig. 6a, b). Consistent with the immunostaining results, flow cytometry analysis of tumor-infiltrating lymphocytes (TILs) showed that anti-DKK2 antibody treatment increase the percentage of GZMB+ T cells (Fig. 6c). NK cells and CD8+ T lymphocytes are the cytotoxic immune cells that are capable of directly killing tumor cells through GrB/perforin pathway^36,37^. Although there were no differences between anti-DKK2-treated and isotype-treated samples in terms of the percentages of infiltrating TILs, CD8a+ T, and NK+ cells (Fig. 6d, e), we observed increased numbers of GZMB+NK+ cells in anti-DKK2-treated tumor samples compared with that in IgG-treated samples (Fig. 6f). These results indicate that the cytotoxic NK cells have significant roles in DKK2 blockade-mediated suppression of tumor formation. Clearly, DKK2 antibody could directly enhance activation of mouse NK cells.

### Applicability of DKK2 blockade for treatment of *Kras*^*LSL-G12D*^; *Apc*^*fl/fl*^ mouse lung cancer

**Fig. 7:**
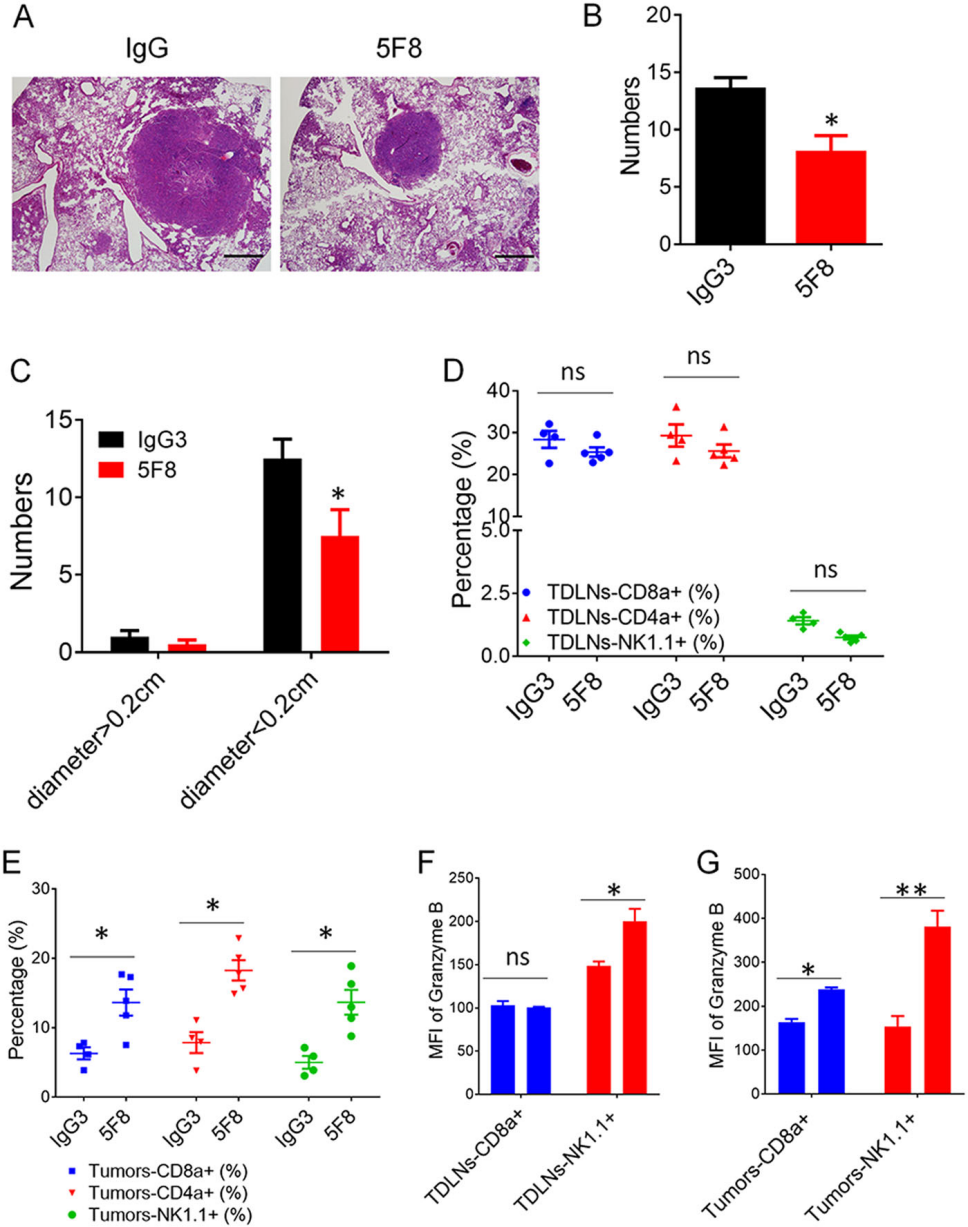
Anti-DKK2 antibody (5F8) suppresses tumor progress and activate immune effector cells in lungs of *Kras*^*G12D*^; *Apc*^*−/−*^ mice. **a**–**c** The mice (8 weeks old) were instilled intranasally with 65 μl of MEM containing adenovirus expressing Cre (2 × 10^7^ plaque-forming unites) and CaCl_2_. After 6 weeks, the mice were treated weekly with IgG or 5F8 (10 mg/Kg). After another 6 weeks, some of the lungs (*n* = 4 IgG and *n* = 4 5F8) are sectioned, and five histological sections from a lung of a representative IgG or 5F8-treated lung are also shown in **a**. Scale bars, 100 μm. Quantification of total tumor number were shown in **b**, and comparison of tumor number in IgG or 5F8 groups after separating tumor as big tumors and small tumors based on tumor diameter (0.2 cm) (**c**). (Data are presented as means ± sem; two-sided Student’s *t* test). **d**–**f** The rest of the lungs (*n* = 4 IgG and *n* = 5 5F8) were analyzed by flow cytometry (**e**, **g**). **d**, **f** Flow cytometry analysis of tumor draining lymph nodes. MFI, mean fluorescence intensity. Data are presented as means ± sem (**p < 0.01 versus IgG; *n* = 4–5; two-sided Student’s *t* test)

Then, we tested DKK2 blockade in a mouse lung cancer model of carrying an activating *Kras* mutation and loss of *Apc*. Treatment of these mice with anti-DKK2 antibody significantly reduced tumor number and tumor size (Fig. 7a–c), and enhanced activation of NK cells in both of tumor draining lymphoid node and tumor tissues (Fig. 7f, g). In addition, we observed that the percentage of infiltrating CD8+ T, NK+, CD4+ T cells also increased in anti-DKK2 antibody treated tumor (Fig. 7e), but no difference in tumor draining lymphoid node (Fig. 7d). The activation of infiltrating CD8+ T cells was also enhanced by anti-DKK2 antibody administration (Fig. 7g). These results, together with the presence of upregulated DKK2 expression in human NSCLCs with *Apc* mutation, and the correlation of high DKK2 expression with poor survival rates revealed by the TCGA suggest that DKK2 blockade may be applicable approaches for NSCLC cells harboring mutations of *APC*.

## Discussion

In this study, we demonstrated that upregulation of DKK2 expression is present in both human NSCLC with *APC* mutation and mice lung tumors caused by *Apc* loss. We uncovered that increased DKK2 promoted lung cancer progression by modulating tumor immune microenvironment. By applying DKK2 monoclonal antibody 5F8, we observed that the 5F8 antibody had a strong tumor-suppressing effect in both orthotopic tumor model and genetically engineered mouse model of lung cancer. This study provides a novel therapeutic approach for NSCLC with *APC* mutation.

Wnt signaling pathways control a wide range of cellular processes^16^. Dysregulation of Wnt-β-catenin signaling has been found contributing to many human cancers^16,17^. DKK2, a Wnt antagonist, inhibits Wnt-β-catenin signaling by binding to LRP6 or LRP5. Aberrant expression of DKK2 has been detected in many tumor tissues^38^. Several epigenetic alterations leading to a decline in the expression of DKK2 in tumors have been reported. For example, frequent epigenetic silencing of DKK2 has been found in ovarian carcinoma^39^, renal cancer^40^, and hepatocellular carcinoma^41^. However, increased DKK2 expression can promote proliferation and invasion via the Wnt signaling in prostate cancer^42^, Ewing sarcoma^43^, and colorectal cancer^44^. DKK2 not only regulates tumor cell proliferation, invasion, and apoptosis directly^40,42,43^, but also modify tumor microenvironment such as angiogenesis^45^. Our recent studies also showed that DKK2 promotes tumor progression by suppressing cytotoxic immune cell activation in colorectal carcinoma with *APC* mutations^29^. In the current study, we expanded our findings of DKK2 functions to human NSCLC with *APC* mutations. Our studies indicated that DKK2 plays similar roles in both colorectal and lung cancer when they carry *APC* mutants. Recently developed therapies targeting signature tumor driving events are largely applicable across tissues-of-origin. For example, Herceptin was originally designed to treat HER2-amplified breast cancer, but later was also approved for HER2-positive metastatic stomach cancer. Anti-PD1/PD-L1 showed durable effects on high mutation burden tumors across multiple cancer types. Recently, larotrectinib was granted approval for the treatment of solid tumors that have a neurotrophic receptor tyrosine kinase gene fusion, regardless of tissues-of-origin. As we showed previously and in this study, DKK2 antibody suppresses tumor growth in both *APC* mutant NSCLC and *APC* mutant colorectal cancer. It is still unknown whether DKK2 plays similar roles in other cancer types with reoccurring *APC* mutations, such as pancreatic cancer. Such studies will further extend applications of DKK2 blockade therapies.

Immunotherapy has become the fourth pillar for various cancer^46,47^. Although the currently approved checkpoint inhibitors including anti-PD1/PD-L1 and anti-CTLA4 have shown clinical efficacy for some tumors, few checkpoints can be counted on our finger^46,47^. In patients with NSCLC, only a small proportion of patients responded to monotherapy of anti-PD-1/PD-L1. Primary and acquired resistance to anti-PD-1/PD-L1 therapies that leads to failure of the therapy has blocked most of the patients from getting clinical benefit^48^. Large effects are being made to develop combinational strategies to overcome resistance to checkpoint inhibitors, majorly in cancer types that are considered high immunogenic, such as melanoma, NSCLC, and colorectal cancer. Recently, we have identified DKK2 as a novel tumor immune-suppressive protein in the microenvironment in colorectal cancer carrying *APC* mutation. We found that DKK2 provided resistance to PD-1 blockade in mouse models of colon cancer^29,49^. We then developed an anti-DKK2 antibody for the immunotherapeutic treatment of colorectal cancer with *APC* mutaions^29,49^. Combinational blockade of DKK2 and PD-1 outperformed on suppression of tumor growth than single antibody treatment in *APC* loss colon tumors^29,49^. *APC* and *KRAS* mutations frequently co-occur in NSCLC. In this study, we found in NSCLC, *APC* mutations drive high expression of Dkk2. DKK2 blockade introduced by anti-DKK2 antibody suppressed tumor growth and enhanced the effects of cytotoxic immune cells in *APC* mutant NSCLC, in consistent with the previous study in colorectal cancer^29^. While mechanisms for loss of *APC* and tumor suppressor *PTEN* regulate DKK2 have been described in colorectal cancer and melanoma^29^, there are more mechanisms in lung cancer yet to be discovered. These possibilities and the therapeutic potential of Dkk2 plus PD-1/PD-L1 blockade in PDX humanized mice model need warrant future investigations.

## Methods

### Mice

*Kras*^*LSL-G12D*^ (*B6.129S4-Krastm4Tyj/J*), and *Apc*^*fl/fl*^ (*C57BL/6-Apctm1Tyj/J*) mice were acquired from the Jackson Laboratory. Wild-type *C57BL/6* mice were purchased from Envigo (Harlan). Mice were housed in specific-pathogen-free conditions and cared for in accordance with the US National Institutes of Health guidelines, and all procedures were approved by the Yale University Animal Care and Use Committee.

### Gene-suppression studies using shRNA

The shRNAs were cloned into pLKO.1-puro lenti-viral vector (Addgene). Viral packaging and infection of cells were performed as previously described^50,51^. After viral infection, cells were selected with puromycin to generate stable cell lines. At least two batches of stable cell lines were generated for each experiment. Experiments were performed in triplicates and repeated at least twice using each batch of cells. The target sequences are:

5′-CAACAAGATGAAGAGCACCAAC-3′ (shScram),

5′-CTGCAAACCAGTGCTCCATCAG-3′ (shDKK2–1),

5′-GCGGGCCAAACTCAACTCCATC-3′ (shDKK2–2),

5′-ACTCCAAGATGCCTCATATAAA-3′ (shDKK2–3).

### Quantitative RT-PCR

Total RNA was prepared and retrotranscribed as described^50,51^. The RT-PCR primers used are: mouse DKK2: 5′-TCAACTCCATCAAGTCCTCTC-3′ (forward) and 5′-TCACATTCCTTATCACTGCTG-3′ (reverse); mouse ACTIN: 5′-cctagaagcatttgcggtgg-3′ (forward) and 5’-gagctacgagctgcctgacg-3′ (reverse).

### Histology

Murine organs were fixed in phosphate-buffered 4% formaldehyde and embedded in paraffin. Three to five micrometres thick sections were stained with hematoxylin and eosin.

Immunofluorescence staining on 5-μm paraffin sections using antibodies against cleaved caspase3 (Cell Signaling, #9664), Goat anti-Rabbit IgG (H + L) Cross-Adsorbed Secondary Antibody, Alexa Fluor 568 (ThermoFisher, #A-11011), and FITC anti-human/mouse Granzyme B Antibody (BioLegend, #515403) was performed as described^8,51,52^.

### Orthotopic tumor model

LLC Lewis lung carcinoma cells (0.5 × 10^6^) were mixed with BD Matrigel (Matrix Growth Factor Reduced) (BD, #354230) in 100 μl and injected subcutaneously into the right flanks of the backs of female C57/BL mice (8–10 weeks old). Tumor growth was measured with calipers, and size was expressed as one-half of the product of perpendicular length and square width in cubic millimeters. For antibody treatment, control IgG3 antibody and anti DKK2 were diluted in PBS, and 100 μl (250 μg) was injected intraperitoneally (i.p.) at the intervals indicated in the figures. For survival tests, mice were euthanized when the tumor size exceeded 1000 mm^3^.

### Flow cytometry

Cells in single-cell suspension were fixed with 2% PFA (Santa-Cruz, #sc-281692). After washing with a Flow Cytometry Staining Buffer (eBioscience, #00–4222–26), cells were stained with antibodies for cell-surface marker for 1 h on ice in the dark. For staining of intracellular proteins, the cells were washed and resuspended in the permeabilization buffer (BD, #554723) and stained by antibodies in the permeabilization buffer for 1 h on ice in the dark. The cells were then pelleted and resuspended in the Flow Cytometry Staining Buffer for flow cytometry analysis. Antibodies used for flow cytometry include: V450 Mouse Anti-Mouse CD45.2 (BD Horizon, #560697, Clone 104), APC anti-mouse NK1.1 Antibody (BioLegend, #108710, clone PK136), FITC anti-human/mouse Granzyme B Antibody (BioLegend, #515403, clone GB11), CD8a Monoclonal Antibody (53–6.7) PE-Cyanine7 (eBioscience, #25–0081–82, clone 53–6.7), CD4 Monoclonal Antibody (RM4–5), and PE (eBioscience, #12–0042–82, clone RM4–5).

### In situ hybridization

In situ hybridization detection of DKK2 mRNA was carried out using the following reagents acquired from Advanced Cell Diagnostics, Inc., based on provided protocol: RNAscope® Target Retrieval Reagents (Cat #322000), RNAscope® Pretreat Reagents- H202 and ProteasePlus (Cat #322330), RNAscope® 2.5 HD Detection Reagent- RED (Cat #322360), RNAscope® Wash Buffer Reagents (Cat #310091), BioCare EcoMount (Cat #320409), ImmECatdge^TM^ Hydrophobic Barrier Pen (Cat #310018), and the mouse Dkk2 probe (#404841).

### Mouse model of lung cancer

*Kras*^*LSL-G12D*^ (*B6.129S4-Krastm4Tyj/J*), and *Apc*^*fl/fl*^ (*C57BL/6-Apctm1Tyj/J*) mice were back crossed to *C57/Bl* background three generations, then *Kras*^*LSL-G12D*^ mice were crossed with *Apc*^*fl/fl*^ mice. For de novo lung cancer mice model, 8-week-old *Kras*^*LSL-G12D*^; *Apc*^*fl/fl*^ mice were treated with 2 × 10^6^ plaque-forming unites of adeno-Cre at 6–8 weeks of age as previously described^8,51,52^. Six weeks later, *Kras*^*LSL-G12D*^; *Apc*^*fl/fl*^ mice were randomly divided into two groups (8–9 for each group) for intraperitoneal injection with either control IgG antibody or anti-DKK2 antibody every other day for another 6 weeks. Four pairs of mice were used for gross inspection and histopathological examination, and the rest of mice in each group were used for flow cytometry analysis.

### Statistical analysis and study design

Minimal group sizes for tumor progression studies were determined via power calculations with the DSS Researcher’s Toolkit with an *α* of 0.05 and power of 0.8. Animals were grouped unblinded, but randomized, and investigators were blinded for the qualification experiments. No samples or animals were excluded from analysis. Assumptions concerning the data including normal distribution and similar variation between experimental groups were examined for appropriateness before statistical tests were conducted. Comparisons between two groups were performed by unpaired, two-tailed *t*-test. Comparisons between more than two groups were performed by one-way ANOVA, whereas comparisons with two or more independent variable factors were performed by two-way ANOVA followed by Bonferroni’s post hoc correction using Prism 6.0 software (GraphPad). Statistical tests were done with biological replicates. *P* < 0.05 was considered statistically significant.

## Supplementary information

Supplementary Information
