# Supplementary material for: Neutralizing monoclonal antibody against Dickkopf2 impairs lung cancer progression via activating NK cells
Source: Cell Death Discov. 2019 Jul 31;5:123. doi: 10.1038/s41420-019-0204-4 (PMC6668384; doi:10.1038/s41420-019-0204-4)
Supplement: Supplementary file 1 — Supplementary Information [file 41420_2019_204_MOESM1_ESM.pdf]

Supplementary Information

Neutralizing monoclonal antibody against Dickkopf2 impairs lung cancer progression via activating NK cells

Authors： Tianli Shen[1,2]*, Zhengxi Chen[2,3]*, Ju Qiao[4], Xuejun Sun[1#] and Qian Xiao[2#]

1. Department of General Surgery, First Affiliated Hospital of Xi’an Jiaotong University, Xi’an, Shaanxi Province, China
2. Department of Pharmacology, School of Medicine, Yale University, 10 Amistad St, New Haven, CT, USA
3. Department of Orthodontics, Shanghai Ninth People′s Hospital, School of Stomatology, Shanghai key Laboratory of Stomatology, Shanghai Jiao Tong University, Shanghai, China
4. Department of Mechanical and Industrial Engineering, Northeastern University, Boston, MA, USA

Correspondence: Xuejun Sun (sunxy@mail.xjtu.edu.cn), and Qian Xiao (qian.xiao@yale.edu)

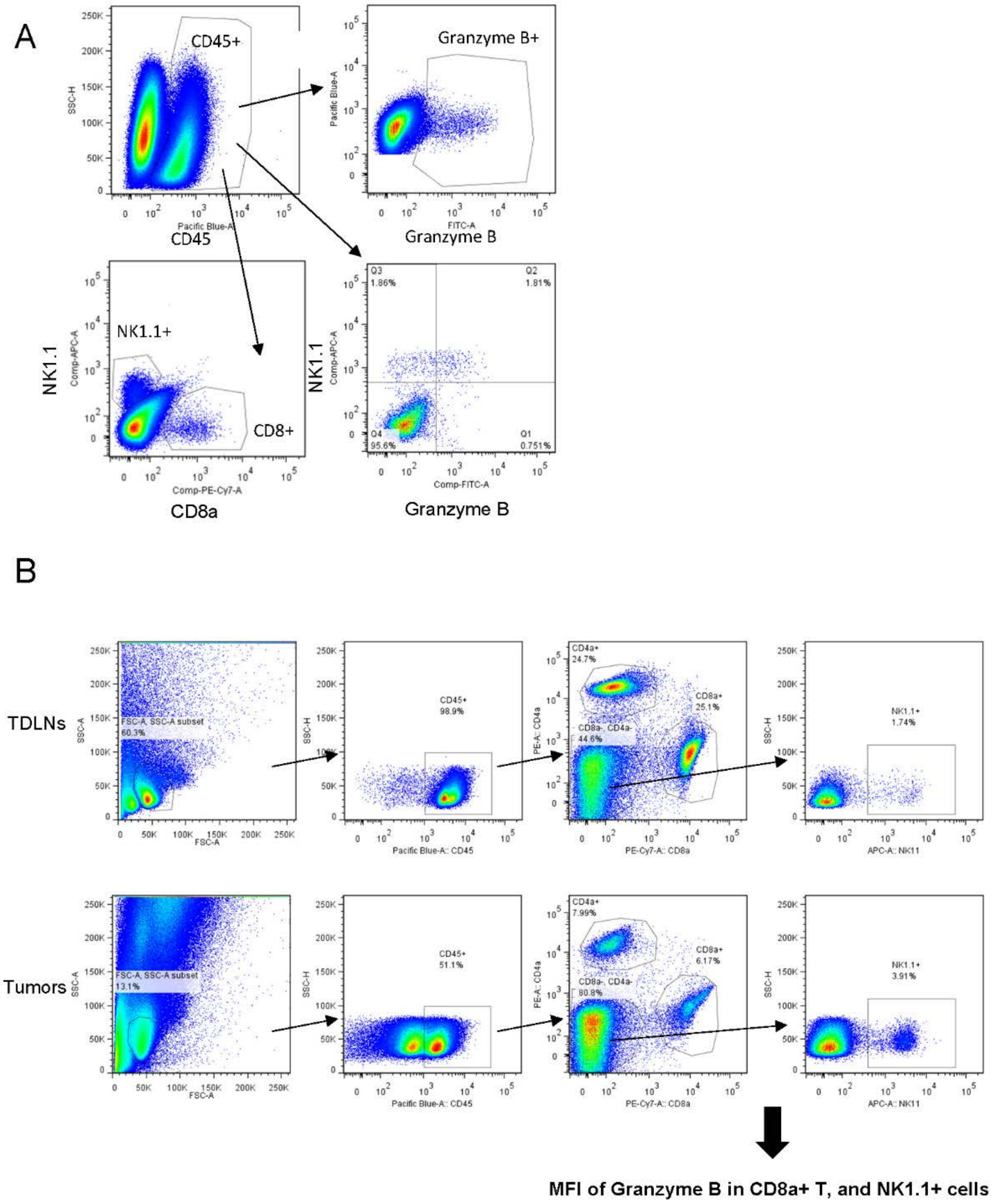

Supplementary Figure 1. Flow cytometry gating strategy.

(A) Flow cytometry gating strategy for Figure 6: CD45+ cells were gated from total tumor cells, then GB+ cells or NK1.1+/CD8a+ cells or NK1.1+/GB+ cells were gated from CD45+ population, respectively. (B) Flow cytometry gating strategy for Figure 7; CD45+ cells were gated from total tumor cells or tumor draining lymph node, then CD4a+ cells or CD8a+ cells were gated from CD45+ population, NK1.1+ cells were gated from CD4a-CD8a- cells. MFI of Granzyme B of CD8a+, and NK1.1+ population were calculated, respectively.
